# BAD-mediated apoptotic pathway is associated with human cancer development

**DOI:** 10.3892/ijmm.2015.2091

**Published:** 2015-02-05

**Authors:** XIAOMANG B STICKLES, DOUGLAS C MARCHION, ELONA BICAKU, ENTIDHAR AL SAWAH, FOROUGH ABBASI, YIN XIONG, NADIM BOU ZGHEIB, BERNADETTE M BOAC, BRIAN C ORR, PATRICIA L JUDSON, AMY BERRY, ARDESHIR HAKAM, ROBERT M WENHAM, SACHIN M APTE, ANDERS E BERGLUND, JOHNATHAN M LANCASTER

**Affiliations:** 1 Department of Women’s Oncology, Cancer Center and Research Institute, Tampa, FL 33612, USA; 2 Chemical Biology and Molecular Medicine, Cancer Center and Research Institute, Tampa, FL 33612, USA; 3 Department of Anatomic Pathology, Cancer Center and Research Institute, Tampa, FL 33612, USA; 4 Department of Oncologic Sciences, Cancer Center and Research Institute, Tampa, FL 33612, USA; 5 Cancer Informatics Core, H. Lee Moffitt, Cancer Center and Research Institute, Tampa, FL 33612, USA

**Keywords:** Bcl-2, carcinogenesis, protein phosphatase 2C, gene expression, reverse transcription quantitative-polymerase chain reaction

## Abstract

The malignant transformation of normal cells is caused in part by aberrant gene expression disrupting the regulation of cell proliferation, apoptosis, senescence and DNA repair. Evidence suggests that the Bcl-2 antagonist of cell death (BAD)-mediated apoptotic pathway influences cancer chemoresistance. In the present study, we explored the role of the BAD-mediated apoptotic pathway in the development and progression of cancer. Using principal component analysis to derive a numeric score representing pathway expression, we evaluated clinico-genomic datasets (n=427) from corresponding normal, pre-invasive and invasive cancers of different types, such as ovarian, endometrial, breast and colon cancers in order to determine the associations between the BAD-mediated apoptotic pathway and cancer development. Immunofluorescence was used to compare the expression levels of phosphorylated BAD [pBAD (serine-112, -136 and -155)] in immortalized normal and invasive ovarian, colon and breast cancer cells. The expression of the BAD-mediated apoptotic pathway phosphatase, PP2C, was evaluated by RT-qPCR in the normal and ovarian cancer tissue samples. The growth-promoting effects of pBAD protein levels in the immortalized normal and cancer cells were assessed using siRNA depletion experiments with MTS assays. The expression of the BAD-mediated apoptotic pathway was associated with the development and/or progression of ovarian (n=106, p<0.001), breast (n=185, p<0.0008; n=61, p=0.04), colon (n=22, p<0.001) and endometrial (n=33, p<0.001) cancers, as well as with ovarian endometriosis (n=20, p<0.001). Higher pBAD protein levels were observed in the cancer cells compared to the immortalized normal cells, whereas PP2C gene expression was lower in the cancer compared to the ovarian tumor tissue samples (n=76, p<0.001). The increased pBAD protein levels after the depletion of PP2C conferred a growth advantage to the immortalized normal and cancer cells. The BAD-mediated apoptotic pathway is thus associated with the development of human cancers likely influenced by the protein levels of pBAD.

## Introduction

The malignant transformation of normal cells may be caused by aberrant gene expression, which disrupts the regulation of cell proliferation, apoptosis, senescence and DNA repair. The Bcl-2 antagonist of cell death (BAD)-mediated apoptotic pathway has been demonstrated to play an important role in carcinogenesis (1) and chemoresponse (2). Evidence suggests that the expression levels of the BAD-mediated apoptotic pathway and BAD protein influence endometrial and ovarian cancer cell resistance to chemotherapy (3,4).

The BAD protein regulates apoptosis by binding to anti-apoptotic members of the same family (5). The BAD protein itself is also regulated by multiple kinases and phosphatases (5–7). When non-phosphorylated, Bad selectively dimerizes with Bcl-xL and Bcl-2, displacing Bax, which is then free to initiate mitochondrial membrane permeability, which leads to apoptosis (8). When phosphorylated, BAD is unable to heterodimerize with Bcl-2 or Bcl-xL and is sequestered into the cytosol by 14-3-3 protein (9). The phosphorylation of 3 serine residues (Ser-112, -136 and -155) influences the activity of the BAD protein. BAD at Ser-112 is phosphorylated by ribosomal protein S6 kinase alpha-1 (RPS6KA1/RSK) and cAMP-dependent protein kinase [also known as protein kinase A (PKA)]; BAD at Ser-136 is phosphorylated by protein kinase B (PKB/Akt) (10) and BAD at Ser-155 is preferentially phosphorylated by PKA (5,11,12). Conversely, the activity of a series of phosphatases, including protein phosphatase(PP)1, PP2A and PP2C (PPM1A), as well as calcineurin, has been shown to exert pro-apoptotic effects through the de-phosphorylation of BAD (13).

We and others have demonstrated that the expression of the BAD-mediated apoptotic pathway and the phosphorylation status of the BAD protein influence the chemosensitivity of cancer cells, including ovarian and endometrial cancer cells (3,4,6,14). Furthermore, we have previously demonstrated that ovarian cancer samples from patients categorized as incomplete responders to primary platinum therapy have a decreased expression of the BAD protein Ser-155 phosphatase, PP2C, compared to samples from patients categorized as complete responders (14). In this study, we investigated the influence of the BAD pathway and the expression of PP2C in the development of cancer. It has previously been suggested that the phosphorylation of BAD at Ser-155 may convey an oncogenic potential (15). In the present study, we demonstrate that the expression of the BAD-mediated apoptotic pathway is associated with the development of a variety of human cancers and that phosphorylated BAD (pBAD) isoforms are overrepresented in cancer cells when compared to immortalized normal cells from the same tissues. Furthermore, using ovarian cancer as a model, we demonstrate the expression of PP2C to be decreased in ovarian cancer when compared to normal ovarian epithelial samples and that depletion of PP2C in immortalized normal ovarian cells, as well as cancer cells provides a growth advantage. Our findings suggest that the BAD-mediated apoptotic pathway influences the development of human cancer and that the expression of PP2C may be an important mediator of oncogenic potential.

## Materials and methods

### Cell lines

The cell lines, CRL-1831 (normal colon epithelial cells), HCT-15 (colorectal adenocarcinoma cells), MCF-7 (breast cancer cells), MCF-10A (mammary epithelial cells), MDA-MB-231 (human breast basal epithelial cells), HEC-1A (endometrial adenocarcinoma cells) and IVAN (immortalized normal ovarian surface epithelial cells), were obtained from the American Type Culture Collection (ATCC; Manassas, VA, USA). OVCAR4 cells were a kind gift from Dr Patricia Kruk, University of South Florida. A2780CP ovarian cancer cells were obtained from the European Collection of Cell Cultures (Salisbury, UK). The cells were cultured at 37°C, 5% CO_2_ in RPMI-1640 or Dulbecco’s modified Eagle’s medium (DMEM) containing 10% fetal bovine serum, 0.01% non-essential amino acids, 1% sodium pyruvate, and 1% penicillin and streptomycin. Mycoplasma testing was performed every 6 months in accordance with the manufacturer’s instructions (Lonza, Rockland, ME, USA).

### Development and evaluation of a BAD-mediated apoptotic pathway gene expression signature

Principal component analysis (PCA) was used to derive a BAD-mediated apoptotic pathway gene expression signature with a corresponding ‘pathway score’ to represent an overall gene expression level for the BAD-mediated apoptotic pathway genes (or subsets thereof for datasets generated by the U133A or U95A Affymetrix platforms, as previously described) (4,16). In brief, PCA was applied to each dataset to reduce the data dimension into a small set of uncorrelated principal components, which were generated based on their ability to account for the systematic variation in the data. In the PCA model, the X matrix (gene expression values) can be described as follows: X = t_1_^*^p_1_′ + t_2_^*^p_2_′ + t_3_^*^p_3_′ + … + t_A_^*^p_A_′ + E (where t_i_ represents scores, p_i_ represents loading and E represents the residual matrix). The scores, t_i_, show how similar samples are to each other, and the loading, p_i_, explains which variables (genes) are important for the principal component *i*. The first principal component (PC1), which accounts for the largest variability in the data, was used as the BAD-mediated apoptotic pathway PCA score to represent the overall expression profile for the BAD-mediated apoptotic pathway. It is known that directional signs of PCA scores are recognized to be arbitrary and can vary between software and algorithm used to calculate the PCA model (17). However, this does not affect the interpretation of the PCA model and can be easily solved by multiplying both scores and loadings by −1, a 180° rotation. Reflecting this, and for the sake of consistency, in our analyses, the PCA model was rotated so a high score corresponded to a normal sample or to an increased survival time.

To evaluate the influence of the BAD-mediated apoptotic pathway on the development and progression of cancer, the BAD-mediated apoptotic pathway PCA score was tested in a series of 6 publically available Affymetrix, U133 Plus GeneChip mRNA expression array datasets from a total of 427 patient samples as follows: i) ovarian samples [28 normal samples, 78 cancer samples, Moffitt Cancer Center (MCC)]; ii) breast samples (143 normal samples, 42 cancer samples, GSE10780); iii) breast samples (8 atypical ductal hyperplasia samples, 23 ductal carcinoma *in situ* samples and 30 invasive ductal carcinoma samples, MCC); iv) colon samples (10 normal samples, 12 cancer samples, GSE4107); v) endometrial samples (9 normal samples, 4 endometrial hyperplasia samples and 20 endometrial cancer samples, MCC); and vi) endometrial samples (10 normal samples, 10 ovarian endometriosis samples, GSE7305). Gene expression data from the normal, pre-invasive and invasive cancer samples for each tissue type were subjected to background correction and normalization using the MAS5 algorithm (Affymetrix Expression Console). The BAD-mediated apoptotic pathway score was evaluated between the normal and cancer, normal and hyperplasia, and carcinoma *in situ* and cancer samples, where available.

### Patients

Patient samples and molecular and clinical data were accessed from the MCC Total Cancer Care (TCC^®^) repository. Patients enrolled in the TCC Protocol had provided written informed consent, and sample collection was approved by the University of South Florida Internal Review Board.

For the ovarian cancer samples, inclusion criteria included age >18 years, ovarian, fallopian, or primary peritoneal cancer, stage III and IV, serous, clear cell, endometrioid, or mixed histology not containing mucinous type. Retrieved clinical data included age at diagnosis, FIGO stage, tumor grade, histological type, surgical cytoreductive status, response to chemotherapy and survival (Table I). Of the total number of patients (n=67), 62 had ovarian, 4 had primary peritoneal and 1 had fallopian tube cancer, with 64 patients undergoing primary debulking (43 optimal, 17 sub-optimal, based on FIGO criteria of residual disease <1 cm, and 4 unknown). The remaining 3 patients received neoadjuvant chemotherapy followed by optimal interval debulking. Of the 9 patients who failed to complete at least 2 cycles of adjuvant chemotherapy, 2 were due to patient refusal, 3 due to death in the post-operative period, 1 patient died during the second cycle, 1 had borderline pathology, 1 stopped due to intolerance and 1 had the disease confined to the ovary (although she was incompletely staged). The 38 patients having a complete response included 3 patients who received neoadjuvant chemotherapy. Complete response was defined by normalized CA-125 post-treatment, disappearance of measurable disease on a CT scan, or negative second-look surgery. Incomplete response (in 17 of our patients) was defined as partial response (decrease in tumor size on a CT scan or decrease but not normalization of CA-125), stable disease (by CT), progression during treatment, or positive second-look surgery. The demographic and baseline data for the patients are listed in Table I.

For the normal ovarian surface epithelial (NOSE) samples, the surface epithelium was carefully scraped from a series (n=9) of normal ovaries that had been resected from patients undergoing surgery for non-ovarian pathology.

### Sample preparation

Macro-dissection was employed to ensure >80% tumor content. Total RNA extraction was performed using the RNeasy mini kit (Qiagen Inc., Hilden, Germany) per manufacturer’s instructions. Fifty nanograms of total RNA were converted into cDNA using TaqMan High Capacity RNA to cDNA master mix (Applied Biosystems/Life Technologies, Grand Island, NY, USA). The following parameters were used for reverse transcription: 30-min hold at 16°C, 30-min hold at 42°C and 5-min hold at 85°C.

### Reverse transcription-quantitative polymerase chain reaction (RT-qPCR)

The NOSE (n=9) and ovarian cancer (n=67) samples were subjected to quantitative (real-time) PCR (RT-qPCR), performed using the relative standard curve method for quantification of relative PP2C expression (StepOne Plus™ real-time PCR system, TaqMan™ small RNA assay and TaqMan Universal PCR master mix; Applied Biosystems/Life Technologies). Glyceraldehyde 3-phosphate dehydrogenase (GAPDH) was used as an endogenous control to normalize the RT-qPCR data. A total of 2 *μ*l of cDNA was used for each RT-qPCR reaction. The following parameters were used for thermocycling: 10 min hold at 95°C, followed by 40 cycles of 15 sec at 95°C, and 60 sec at 60°C. Analysis was performed using StepOne software (version 2.1). The expression level of PP2C in an ovarian cancer cell line, SKOV6 (kind gift from Dr Susan Murphy, Duke University), was used as the reference point (relative expression level of 1), to which all patient samples were compared.

### Transfection and assessment of cell proliferation

Transfection was performed through electroporation using the Amaxa Nucleofactor II™ (Lonza). Cells (4×10^6^) were transfected with either PP2C siRNA or a non-targeting negative control siRNA (Applied Biosystems/Life Technologies), with a final siRNA concentration of 1 *μ*M. Following transfection, the cells were seeded in 96-well optical plates. Subsequent proliferation was measured via MTS assay using CellTiter 96 Aqueous One Solution (Promega, Madison, WI, USA) at 24-h intervals. Baseline proliferation at 24 h post-transfection was used to normalize subsequent assays. The proliferation of the cells transfected with PP2C siRNA was expressed as a percentage of the proliferation of the cells transfected with non-targeting negative control siRNA.

### Immunofluorescence microscopy

Immortalized normal and invasive cancer cell lines of different cancer types, such as ovarian, colon, breast and endometrial cancer, were cultured using standard techniques. Twenty thousand cells were plated in each well of standard 12-well plates for 24 h. The cells were then fixed with a solution of 95% ethanol and 5% acetic acid for 1 min. The cells were washed 3 times with phosphate-buffered saline (PBS) and then incubated with 2% bovine serum albumin (BSA) in PBS. Primary antibody was added to a blocking serum consisting of 2% BSA in PBS for 24 h. After 5 washes, the cells were incubated with fluorescent-labeled secondary antibody in blocking serum for 1 h. The wells were then counterstained and mounted with Prolong Gold containing 4′,6-diamidino-2-phenylindole (DAPI) (Invitrogen/Life Technologies, Grand Island, NY, USA). Fluorescence images were obtained using the AxioCam MRm CCD camera and AxioVision (version 4.7). Exposure times were identical for each antibody across the cell line pairs. The intensity of fluorescence for each image was determined using Definiens Developer XD 1.5 software. An algorithm was developed to extract the fluorescence intensity per cell. The immunofluorescence intensity of pBAD was expressed as a proportion of the intensity of total BAD for n=50 cells of each cell line. The immortalized normal cell line was then set as the reference to which its paired cancer cell line was compared. Primary antibodies included rabbit anti-phospho-BAD[Ser-112] (#A0029) and rabbit anti-phospho-BAD[Ser-136] (#A01156) were acquired from Genscript (Piscataway, NJ, USA). Rabbit anti-phospho-BAD[Ser-155] (#9297) and rabbit anti-pan-BAD (#9292) were acquired from Cell Signaling Technologies (Danvers, ME, USA). Secondary antibody included goat anti-rabbit AlexaFluor 546 (#A11010) was obtained from Invitrogen/Life Technologies.

### Western blot analysis

The cells were harvested in medium and washed with cold PBS containing 1X phosphatase inhibitor cocktail (Sigma-Aldrich, St. Louis, MO, USA). Lysates were prepared with sodium dodecyl sulfate (SDS) lysis buffer (2% SDS, 10% glycerol, 0.06 M Tris; pH 6.8) and evaluated for protein concentration using the bicinchoninic acid method (Pierce, Rockford, IL, USA). Proteins (75 *μ*g) were separated on the same day as collection time on 12–15% SDS-polyacrylamide gel (PAGE) gels and transferred onto polyvinylidene fluoride membranes. The membranes were blocked with 5% non-fat milk in Tris-buffered saline containing 0.05% Tween-20 (TBST) and incubated with primary antibody in 5% non-fat milk in TBST overnight at 4°C. The membranes were washed 3 times for 5 min with TBST and incubated with the appropriate secondary antibody in 5% non-fat milk in TBST for 60 min at room temperature. The membranes were washed 4 times for 5 min with TBST prior to antibody binding visualization using SuperSignal West Pico chemiluminescence solution (Pierce) on autoradiography film (Midwest Scientific, St. Louis, MO, USA). Primary antibodies included rabbit anti-phospho-BAD[Ser-155] (#9297; Cell Signaling Technologies), mouse anti-PP2C (#Sc-56956; Santa Cruz Biotech, Santa Cruz, CA, USA), and mouse anti-GAPDH (#MAB374; Millipore, Temecula, CA, USA). Secondary antibodies included donkey anti-rabbit-HRP (#NA9340V) and sheep anti-mouse-HRP (#NA931V) from GE Healthcare, Pittsburgh, PA, USA.

### Statistical analysis

Differences in BAD pathway expression as defined by the PC1 score as well as differences in percentage cell growth were evaluated by the Student’s t-test. A p-value ≤0.05 was considered to indicate a statistically significant difference. Spearman’s correlation was used to evaluate differences in BAD pathway expression between normal, hyperplasia and cancer samples. A p-value ≤0.05 indicated a significant difference.

## Results

### BAD-mediated apoptotic pathway expression is associated with the development of cancer

**Figure 1:**
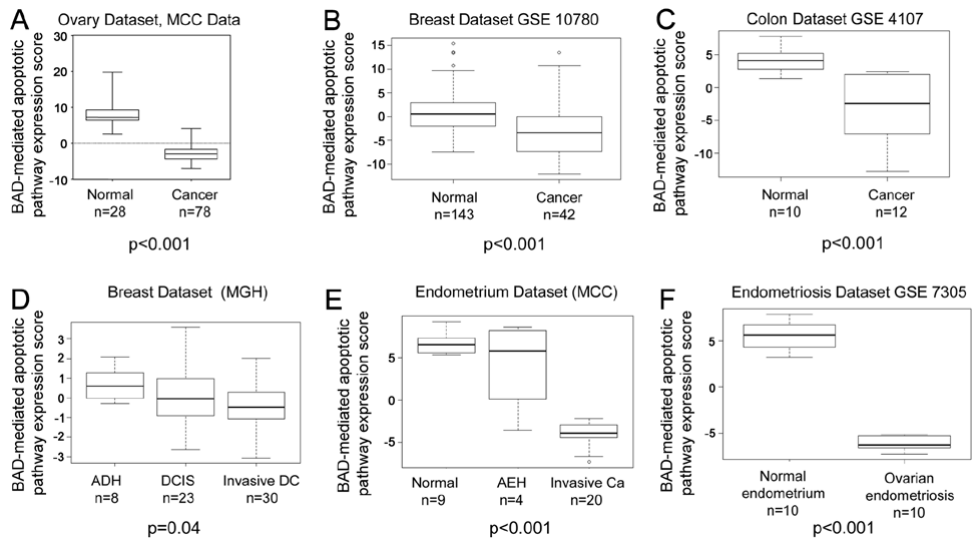
Expression of the BAD-mediated apoptotic pathway is associated with the development and progression of cancer. BAD-mediated apoptotic pathway expression, as modeled by principal component analysis (PCA), was higher in the corresponding normal tissue samples than in the (A) ovarian cancer samples (p<0.001); (B) breast cancer samples (p<0.001); and (C) colon cancer samples (p<0.001). When BAD-mediated apoptotic pathway expression was compared among various stages of cancer progression, the expression score was higher in (D) breast tissue samples, with atypical ductal hyperplasia (ADH) > ductal carcinoma *in situ* (DCIS) > invasive ductal carcinoma (IDC) (p=0.04); in (E) endometrial cancer tissue samples, with normal > atypical endometrial hyperplasia (AEH) > invasive carcinomas (p<0.001); and in (F) endometriosis tissue samples, with normal > ovarian endometriosis samples (p<0.001).

In each tissue type examined, the BAD-mediated apoptotic pathway PCA score was higher in the normal tissue than in the corresponding invasive carcinoma samples (Fig. 1). The normal ovary samples (n=28) had a mean BAD-mediated apoptotic pathway expression score of 8.1968, whereas the ovarian cancer samples (n=78) had a mean score of −2.9424 (P<0.001) (Fig. 1A). The normal breast samples (n=143) had a mean BAD-mediated apoptotic pathway expression score of 2.721 vs. a score of −0.799 for the breast cancer samples (n=42; P<0.001) (Fig. 1B). The normal colon samples (n=10) had a mean BAD-mediated apoptotic pathway expression score of 4.049 vs. a value of −3.374 for the colon cancer samples (n=12; P<0.001) (Fig. 1C). When the BAD-mediated apoptotic pathway expression results were compared among the various stages of cancer progression, the expression score was higher in the atypical ductal hyperplasia breast tissue samples (mean expression score, 0.687, n=8) than in the ductal carcinoma *in situ* samples (mean expression score, 0.046, n=23) and higher in ductal carcinoma *in situ* than in invasive ductal carcinoma (mean expression score, −0.298, n=30, Spearman’s correlation estimate, −0.264, P=0.04) (Fig. 1D). BAD-mediated apoptotic pathway expression was higher in the normal endometrial samples (mean expression score, 6.745, n=9) than in the hyperplastic tissue samples (mean expression score, 4.161, n=4) and higher in the hyperplastic tissue samples than in the carcinoma samples (mean expression score, −3.867, n=20, Spearman correlation estimate, −0.795, P<0.001) (Fig. 1E). The mean BAD-mediated apoptotic pathway expression score of the normal endometrium samples (n=10) was 5.614 vs. −5.614 in the ovarian endometriosis samples (n=10, P<0.001) (Fig. 1F).

### BAD phosphorylation status and cancer development

**Figure 2:**
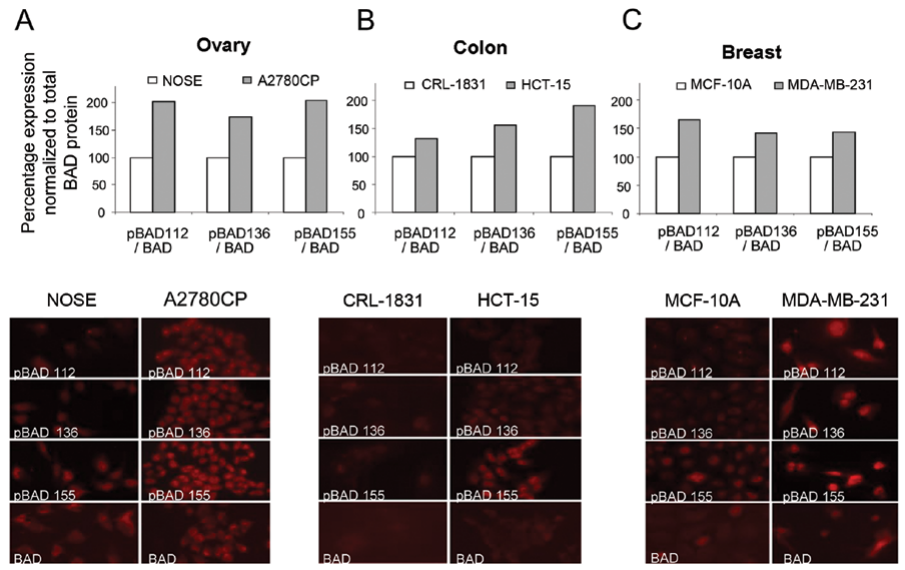
Phosphorylated BAD (pBAD) isoforms are expressed at higher levels in cancer cells. Immortalized normal and cancer cell line pairs were evaluated for pBAD (serine-112, -136 and -155) levels by immunofluorescence. Image analysis of fluorescence intensity indicated that cancer cells of the (A) ovary, (B) colon, and (C) breast expressed higher percentages of pBAD (serine-112, -136 and -155) to total BAD protein than the paired immortalized normal cell line. Bar graphs show pBAD-to-total BAD ratios of at least 50 cells per condition depicted as the percentage expression in cancer cells relative to immortalized normal cells. NOSE, normal ovarian surface epithelial.

The post-translational modification of BAD represents a key control point in the determination of cell survival vs. apoptosis (5,19). Previously, we demonstrated an inverse correlation between the phosphorylation status of the BAD protein and BAD-mediated apoptotic pathway PCA score (4). In light of this and the identified differences in BAD-mediated apoptotic pathway expression between normal and cancer tissues, we evaluated differences in BAD phosphorylation between normal and cancer cells. We analyzed the expression levels of pBAD (Ser-112, -136 and -155), as well as total BAD, by immunofluorescence, comparing a normal (immortalized) cell line with a cancer cell line from several tissue types, including ovarian (NOSE vs. A2780CP) (Fig. 2A), colon (CRL-1831 vs. HCT-15) (Fig. 2B) and breast (MCF-10A vs. MBA-231) (Fig. 2C). Compared to the immortalized normal cells, the cancer cell lines showed an increase in the percentage of pBAD relative to the total BAD protein levels.

### PP2C levels are associated with cancer

**Figure 3:**
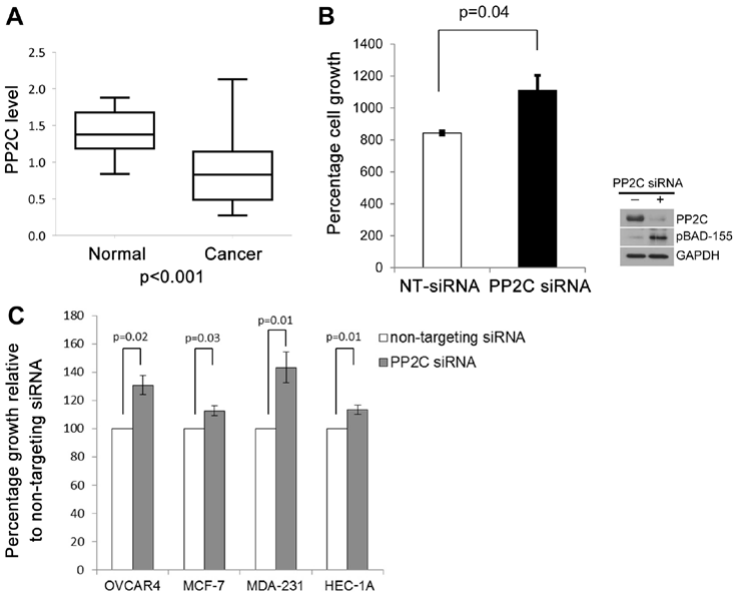
Protein phosphatase 2C (PP2C) expression influences the development and progression of cancer. (A) Ovarian cancer samples (n=67) expressed lower mRNA levels of the BAD-mediated apoptotic pathway phosphatase PP2C than normal ovary samples (n=9), as shown by RT-qPCR (p<0.001). (B) The depletion of PP2C resulted in the increased expression of phosphorylated BAD (pBAD) at Ser-155 (pBAD-155), as shown by western blot analysis, and increased cell growth rates, as shown by MTS assay in the immortalized normal ovary cell line, IVAN. (C) The depletion of PP2C conferred a growth advantage to the cancer cell lines, OVCAR4 (ovarian cancer), MCF-7 (breast cancer), MDA-MB-231 (MDA-231; breast cancer), and HEC-1A (endometrial cancer). Error bars depict the standard error of the mean. Student’s t-test p-values were derived from the average of 3 replicate experiments.

PP2C is a key phosphatase that influences BAD protein phosphorylation status (13). We previously demonstrated that decreased PP2C levels were associated with increased chemoresistance in ovarian cancer and endometrial cancer cells, as well as primary ovarian cancer samples (3,4,14). In this study, to determine whether PP2C levels may play a role in ovarian carcinogenesis, we evaluated the PP2C mRNA levels by RT-qPCR in a dataset of 9 normal ovarian surface epithelial samples and 67 primary ovarian cancer samples. We found that the mean relative expression of PP2C was 0.864 in the ovarian cancer patient samples (95% CI, 0.763–0.965, n=67) and 1.403 (95% CI, 1.188–1.618, n=9) in the normal ovarian epithelial samples (P<0.001) (Fig. 3A). No statistically significant differences were observed between PP2C expression and clinical variables, including response to therapy (complete vs. incomplete response), disease-free survival (long vs. short) and overall survival (Table II). Patients with complete response showed a higher level of PP2C expression than those with incomplete response, and patients who had undergone optimal debulking showed a higher level of PP2C than patients who had suboptimal debulking. However, neither comparison reached statistical significance (Table II).

To further explore a role for PP2C in human cancer development, we evaluated whether decreased PP2C levels may provide a growth advantage to normal ovary epithelial cells. We evaluated the cell growth rates following the depletion of PP2C by siRNA in the immortalized normal ovarian surface epithelial cells, IVAN. Depletion of PP2C by siRNA in the IVAN cells resulted in increased cell growth rates for up to 5 days when compared to the cells transfected with non-targeting negative control siRNA (Fig. 3B). Increased cell growth rates were accompanied by an upregulation of pBAD, Ser-155 levels (Fig. 3B). To determine whether PP2C levels may influence the progression of cancer, we evaluated the effects of the depletion of PP2C on the growth rates of i) ovarian cancer cells (OVCAR4), ii) breast cancer cells (MCF-7 and MDA-MB-231), and iii) endometrial cancer cells (HEC-1A). Similar to the IVAN cells, the depletion of PP2C by siRNA provided a growth advantage to all cancer cell lines examined (Fig. 3C). As shown in Fig. 3C, a significant increase in cell growth at 72 h after the depletion of PP2C was observed in the cancer cell lines, OVCAR4 (P=0.02), MCF-7 (P=0.03), MDA-MB-231 (P=0.01) and HEC-1A (P=0.01).

## Discussion

The evasion of apoptotic signaling is a hallmark of cancer cells (18). Since Bcl-2 family proteins are critical determinants of cellular apoptosis and survival, we evaluated the role of the BAD-mediated apoptotic pathway as a determinant of cancer development and progression. We previously developed a BAD-mediated apoptotic pathway gene expression signature using PCA that summarized the overall expression of the BAD pathway and found that the expression of the BAD pathway was associated with the development of ovarian cancer chemoresistance (4). In this study, we evaluated whether the BAD-mediated apoptotic pathway also influences carcinogenesis. Using PCA modeling, we evaluated the associations between BAD-mediated apoptotic pathway expression and carcinogenesis using a series of clinico-genomic datasets comprising normal and cancer tissues, including cancers of the breast, colon and endometrium. We revealed differences in BAD-mediated apoptotic pathway expression between the normal tissue and cancer samples. Moreover, we observed a correlation between the BAD-mediated apoptotic pathway expression score and the transition from normal tissue to pre-cancer/pre-invasive cancer, to invasive cancer, suggesting that the BAD-mediated apoptotic pathway influences the development and progression of several solid tumor types.

Bcl-2 family proteins determine cell survival by both differential expression and post-translational modifications. The pro-apoptotic activity of BAD is inhibited by phosphorylation at the Ser-112, -136 and -155 sites. The post-translational modification of BAD represents a key control step between cell survival and apoptosis. Thus, the phosphorylation of these serine residues is required to prevent BAD-induced apoptosis (5,19). We found the percentage of pBAD to be higher in cancer cells of the ovary, breast and colon than corresponding immortalized normal cell lines. Furthermore, we demonstrated that PP2C, a BAD phosphatase at Ser-155, is expressed at a higher level in normal ovaries than in ovarian cancers. The phosphorylation of BAD at Ser-155 is known to contribute to cancer cell survival *in vitro* (20). Our present findings demonstrate that the depletion of PP2C, resulting in increased levels of pBAD at Ser-155, confers a significant growth advantage to both immortalized normal and cancer cell lines. We also observed a trend toward higher *in vivo* levels of PP2C in chemosensitive cancers vs. chemoresistant ovarian cancers, although this did not reach statistical significance. The range of PP2C expression was wider in the ovarian cancer samples than in the normal ovary samples, thus suggesting that additional factors may influence the phosphorylation status of BAD and thus the potential for oncogenesis.

In conclusion, our results suggest that the subversion of BAD-mediated apoptosis may be an important step in human cancer development and progression. It may also be an important mechanism through which cancer cells acquire increased growth potential. Further elucidation of the interactions between various members of the BAD-mediated apoptotic pathway may lead to the identification of novel targets for molecular therapy and biomarker development.

## Figures and Tables

**Table I:** Patient demographics.

Characteristics	n	%
Age at diagnosis (mean, 63 years)		
<45 years	3	4
45–65 years	28	42
>65 years	3	48
Unknown	4	6
FIGO stage		
3	53	79
4	11	16
Other	3	4
Tumor grade		
Low	7	10
Moderate	9	13
High	51	76
BRCA-positive	2	3
Debulking status (n=64)		
Optimal	43	67
Sub-optimal	17	27
Unknown	4	6
Response to chemotherapy		
CR	38	57
IR	17	25
Received <2 cycles	9	13
Unknown	3	4
Histology		
Serous	52	78
Clear cell	2	3
Endometrioid	1	1
Mixed	7	10
Undifferentiated	3	4
Other	2	3

BRCA, breast cancer gene; CR, complete response; IR, incomplete response. Total n=67 (except where noted).

**Table II:** PP2C relative expression in normal ovary and ovarian cancer samples.

Group	Relative mean PP2C expression	95% CI	P-value
Normal ovary	1.403	1.188–1.618	<0.001
All ovarian cancers	0.864	0.763–0.965	
CR	0.867	0.726–1.008	0.413
IR	0.765	0.569–0.961	
Optimally debulked	0.875	0.732–0.963	0.34
Sub-optimally debulked	0.771	0.604–0.938	
Short DFS	0.893	0.724–1.063	0.51
Long DFS	0.819	0.68–0.959	
Short overall survival	0.81	0.676–0.944	0.36
Long overall survival	0.876	0.7–1.052	

PP2C, protein phosphatase 2C; CR, complete response; IR, incomplete response; DFS, disease-free interval.

## References

[1] Marone M, Scambia G, Mozzetti S (1998). bcl-2, bax, bcl-XL, and bcl-XS expression in normal and neoplastic ovarian tissues. Clin Cancer Res.

[2] Llambi F, Green DR (2011). Apoptosis and oncogenesis: give and take in the BCL-2 family. Curr Opin Genet Dev.

[3] Chon HS, Marchion DC, Xiong Y (2012). The BCL2 antagonist of cell death pathway influences endometrial cancer cell sensitivity to cisplatin. Gynecol Oncol.

[4] Marchion DC, Cottrill HM, Xiong Y (2011). BAD phosphorylation determines ovarian cancer chemosensitivity and patient survival. Clin Cancer Res.

[5] Tan Y, Demeter MR, Ruan H, Comb MJ (2000). BAD Ser-155 phosphorylation regulates BAD/Bcl-XL interaction and cell survival. J Biol Chem.

[6] Hayakawa J, Ohmichi M, Kurachi H (2000). Inhibition of BAD phosphorylation either at serine 112 via extracellular signal-regulated protein kinase cascade or at serine 136 via Akt cascade sensitizes human ovarian cancer cells to cisplatin. Cancer Res.

[7] Zha J, Harada H, Yang E, Jockel J, Korsmeyer SJ (1996). Serine phosphorylation of death agonist BAD in response to survival factor results in binding to 14-3-3 not BCL-X(L). Cell.

[8] Yang E, Zha J, Jockel J, Boise LH, Thompson CB, Korsmeyer SJ (1995). Bad, a heterodimeric partner for Bcl-XL and Bcl-2, displaces Bax and promotes cell death. Cell.

[9] Hirai I, Wang HG (2001). Survival-factor-induced phosphorylation of Bad results in its dissociation from Bcl-x(L) but not Bcl-2. Biochem J.

[10] del Peso L, González-García M, Page C, Herrera R, Nunez G (1997). Interleukin-3-induced phosphorylation of BAD through the protein kinase Akt. Science.

[11] Lizcano JM, Morrice N, Cohen P (2000). Regulation of BAD by cAMP-dependent protein kinase is mediated via phosphorylation of a novel site, Ser155. Biochem J.

[12] Zhou XM, Liu Y, Payne G, Lutz RJ, Chittenden T (2000). Growth factors inactivate the cell death promoter BAD by phosphorylation of its BH3 domain on Ser155. J Biol Chem.

[13] Klumpp S, Selke D, Krieglstein J (2003). Protein phosphatase type 2C dephosphorylates BAD. Neurochem Int.

[14] Bansal N, Marchion DC, Bicaku E (2012). BCL2 antagonist of cell death kinases, phosphatases, and ovarian cancer sensitivity to cisplatin. J Gynecol Oncol.

[15] Youle RJ, Strasser A (2008). The BCL-2 protein family: opposing activities that mediate cell death. Nat Rev Mol Cell Biol.

[16] Ma XJ, Salunga R, Tuggle JT (2003). Gene expression profiles of human breast cancer progression. Proc Natl Acad Sci USA.

[17] Jolliffe IT (2002). Principal Component Analysis.

[18] Hanahan D, Weinberg RA (2011). Hallmarks of cancer: the next generation. Cell.

[19] Datta SR, Katsov A, Hu L (2000). 14-3-3 proteins and survival kinases cooperate to inactivate BAD by BH3 domain phosphory-lation. Mol Cell.

[20] Virdee K, Parone PA, Tolkovsky AM (2000). Phosphorylation of the pro-apoptotic protein BAD on serine 155, a novel site, contributes to cell survival. Curr Biol.

